# A user-friendly mathematical modelling web interface to assist local decision making in the fight against drug-resistant tuberculosis

**DOI:** 10.1186/s12879-017-2478-6

**Published:** 2017-05-30

**Authors:** Romain Ragonnet, James M. Trauer, Justin T. Denholm, Ben J. Marais, Emma S. McBryde

**Affiliations:** 1 Faculty of Medicine, Dentistry and Health Sciences, University of Melbourne, Melbourne, Australia; 2 Burnet Institute, 85 Commercial Road, Melbourne, VIC 3141 Australia; 3 Victorian Tuberculosis Program, Melbourne Health, Melbourne, Australia; 4 School of Public Health and Preventive Medicine, Monash University, Melbourne, Australia; 5 Department of Microbiology and Immunology, University of Melbourne at the Peter Doherty Institute, Melbourne, Australia; 6 Victorian Infectious Diseases Service, Royal Melbourne Hospital, Parkville, VIC Australia; 7 Marie Bashir Institute and the Centre for Research Excellence in Tuberculosis, University of Sydney, Sydney, Australia; 8 Australian Institute of Tropical Health and Medicine, James Cook University, Townsville, Australia

**Keywords:** User Interface, Decision making, Tuberculosis, Multidrug-resistant tuberculosis, Re-treatment, Causal pathway, Misdiagnosis

## Abstract

Multidrug-resistant and rifampicin-resistant tuberculosis (MDR/RR-TB) represent an important challenge for global tuberculosis (TB) control. The high rates of MDR/RR-TB observed among re-treatment cases can arise from diverse pathways: de novo amplification during initial treatment, inappropriate treatment of undiagnosed MDR/RR-TB, relapse despite appropriate treatment, or reinfection with MDR/RR-TB. Mathematical modelling allows quantification of the contribution made by these pathways in different settings. This information provides valuable insights for TB policy-makers, allowing better contextualised solutions. However, mathematical modelling outputs need to consider local data and be easily accessible to decision makers in order to improve their usefulness. We present a user-friendly web-based modelling interface, which can be used by people without technical knowledge. Users can input their own parameter values and produce estimates for their specific setting. This innovative tool provides easy access to mathematical modelling outputs that are highly relevant to national TB control programs. In future, the same approach could be applied to a variety of modelling applications, enhancing local decision making.

## The threat of drug-resistant TB

Drug-resistant tuberculosis (TB) is a threat to TB control and a barrier to disease elimination. TB treatment is complicated by resistance to rifampicin and/or isoniazid, the two most active first-line TB drugs. At the global level, 3.9% (95% confidence interval [CI]: 2.7–5.1%) of all new TB cases are multidrug-resistant (defined as resistant to at least rifampicin and isoniazid) or rifampicin-resistant (MDR/RR-TB). However, the proportion of MDR/RR-TB globally is even higher among re-treatment cases, reaching 21% (95% CI: 15–28%) according to estimates by the World Health Organization (WHO) [1]. Mathematical modelling suggests that MDR-TB strains could become dominant over the drug-susceptible (DS) ones in the coming decades [2], threatening the success of WHO’s End TB Strategy and targets for TB elimination. A more comprehensive understanding of the different mechanisms leading to MDR/RR-TB at re-treatment is important to provide enhanced insight into the local determinants of drug-resistant TB emergence and to support the development of better contextualised solutions.

## The pathways to drug resistance at re-treatment

The higher proportion of MDR/RR-TB in re-treatment cases compared to new cases has long been attributed to resistance amplification due to poor treatment adherence, focusing the response to emergent drug-resistant TB on better treatment supervision of patients treated with first-line therapy [3, 4]. Among re-treated cases, finding of MDR/RR-TB rarely triggers consideration of pathways that involve primary transmission of drug-resistant strains, i.e. involving infection with an already drug-resistant pathogen. However, three different causal pathways to MDR/RR-TB at re-treatment can be identified that do not involve resistance amplification and are therefore illustrations of primary transmission. First, the low rate of drug susceptibility testing among new TB cases results in a large proportion of initially drug-resistant cases being treated with inappropriate regimens, leading to treatment failure or disease relapse and re-presentation as a “re-treatment case” [1]. In addition, among new cases correctly diagnosed with MDR/RR-TB, treatment outcomes are often unfavourable, making resistant cases more likely to present for re-treatment. Finally, reinfection with a drug-resistant strain may also contribute to the burden of MDR/RR-TB seen at re-treatment, especially in settings where TB transmission is poorly controlled. We developed a modelling approach to quantify the likely proportions of MDR/RR-TB at re-treatment attributed to the different causal pathways specified [5]. Findings highlighted the failure to identify drug resistance at first presentation as a leading source of MDR/RR-TB among re-treatment cases at the global level. However, when applying our model to different regions and countries, we demonstrated substantial variability in the respective contributions of the various sources at the national and local levels. This finding highlights the need for better contextualised solutions that utilise local data to guide local TB control priorities and interventions.

## The need to make mathematical modelling more applicable and usable

While our previous study provided estimates at the national level for more than 100 countries, detailed assessment of the dominant drug resistance pathways in specific settings would assist local control efforts. Strong spatial heterogeneity in the burden of MDR/RR-TB has been identified in several countries [6–9], with demonstration that transmission can reach extreme intensities in small localities [10], emphasising the need for better contextualised solutions. In such settings, refined estimates incorporating local information would be invaluable in producing realistic and actionable model outputs. Estimates reported in our previous study only provided insight relevant to 2015 data, as reported in the 2016 WHO Global Tuberculosis Report [1]. However, this fails to take account of the changing aspects of the TB epidemic and the need for real-time decision making in order to optimize programmatic responses. Allowing real-time assessment of likely MDR/RR-TB pathways will provide updated estimates as routine data are gathered and refreshed over time. In addition, policy makers may wish to test alternate assumptions or to use parameter values that they believe more appropriate. Such continuity and flexibility cannot be provided by occasional external expert support or traditional research project-based approaches, and thus represent a major barrier to the effective use of mathematical modelling for everyday policy guidance.

To overcome this limitation, present in most modelling outputs reported to date, we developed a user-friendly interface for real-time analysis of local data using our recently published model of MDR/RR-TB pathways [5]. In the current paper we introduce a web-based interactive tool to quantify the likely contribution of different MDR/RR-TB pathways among re-treatment cases, accommodating localised parameterisation via a user-friendly interface.

## The user-friendly modelling interface

### General description

**Fig. 1:**
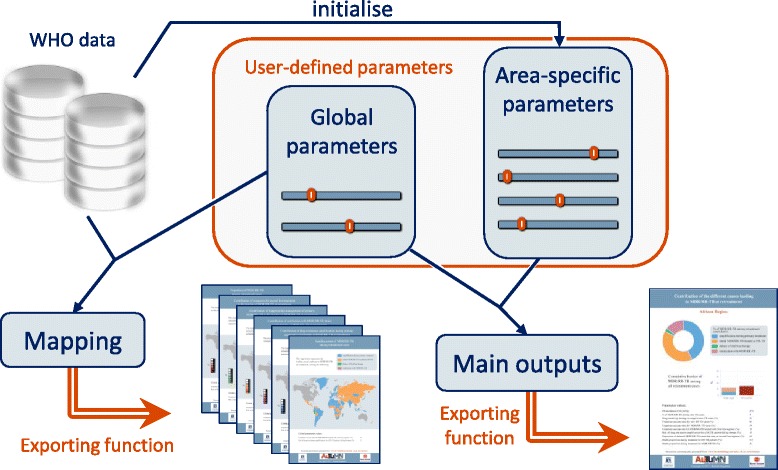
General description of the user interface

The user interface is available online at www.tb-modelling.com/mdr_tb_at_retreatment. Its main objective is to quantify the proportions of MDR/RR-TB at re-treatment attributed to four principle causal pathways: I) initial drug-susceptible TB with resistance amplification during treatment; II) initial MDR/RR-TB inappropriately treated as drug-susceptible TB; III) MDR/RR-TB relapse despite appropriate treatment; and IV) re-infection with MDR/RR-TB. The model employed to estimate these contributions was described in detail in our previous publication [5]. The online tool allows users to produce estimates by using the model in combination with their own inputs. The associated model outputs are produced in real time and exporting features allow the user to download personalised reports in a PDF format. Figure 1 summarises the general principle and the different functionalities of the platform. Javascript language was used to build the interactive platform as we needed immediate responsiveness to produce model outputs instantaneously when users specify new inputs. Thus, the webpage is only loaded once from the remote server and calculations are then performed on the user’s device in real time when parameter values are changed. We used *Chart*, *jVectorMap* and *jsPDF* libraries to generate the charts, maps and exporting functionalities, respectively.

### Personalised inputs

The user must specify the area (WHO region or country) to which the model will apply. This process allows preselected parameter sets to be generated based on local WHO data. While these default values correspond to the ones that were used in the initial study [5], they can here be adjusted using a set of slider bars. Parameters are classified into two main categories: ‘area-specific’ and ‘global’, with only parameters of the first category updated when a new area is selected. Compatibility tests on parameter values are run in the background to ensure that the parameterisation remains realistic. For example, if the user selected a treatment success rate for DS-TB of 80%, a ceiling is set for the death proportion during treatment for DS-TB at 20%.

### Model outputs

**Fig. 2:**
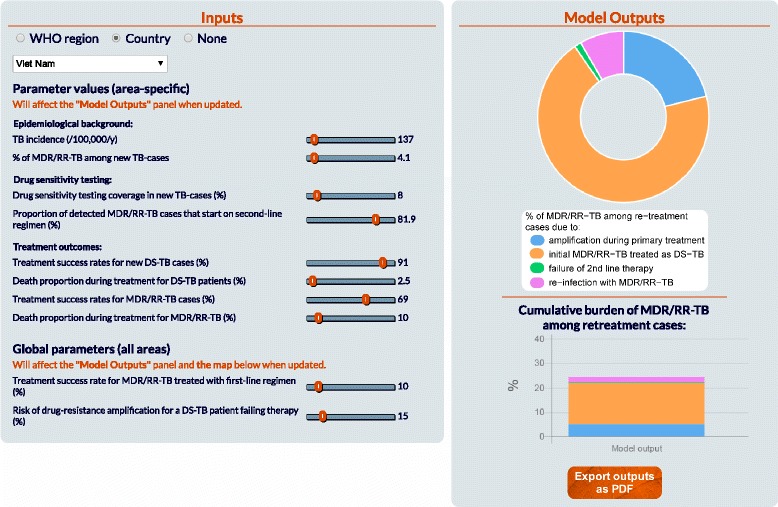
Partial screen capture of the user interface. The *Inputs* panel contains the predefined parameter values associated with Viet Nam. The results displayed in the *Model Outputs* panel are instantaneously updated when any of the parameters is adjusted through the sliders of the *Inputs* panel

The *Model Outputs* panel consists of two charts that are automatically updated when any of the parameter values is modified. First, a doughnut chart represents the proportions of MDR/RR-TB at re-treatment attributed to the four principle causal pathways I-IV. Second, a bar chart displays the proportion of MDR/RR-TB among all re-treatment TB cases. A comparison between the model output and the WHO estimate regarding this proportion is also presented on the second chart when relevant data are available from WHO for the selected area. Quantitative estimates are displayed when the user moves the pointer over the different shares of the two charts. Figure 2 presents a screen capture of the interface containing the *Inputs* and *Model Outputs* panels for an example model estimation.

### Mapping

An interactive world map is incorporated in the user interface to provide a global visualisation of the different country-level estimates. The user can select one of the six model outputs that can be displayed on the choropleth map: individual contribution of Cause I, II, III or IV; leading cause of MDR/RR-TB at re-treatment; and proportion of MDR/RR-TB among all re-treatment TB cases. The results presented on the maps are calculated using the user-defined values for the global parameters and the default values for the area-specific parameters. That is, changes to the area-specific parameter values associated with a selected country will not affect the maps (see Fig. 1). When the user clicks on a country, the *Inputs* panel will be updated and the *Model Outputs* panel will display estimates related to the selected country.

### Download personalised reports

Exporting functionalities are available allowing the users to download PDF documents incorporating the model outputs associated with their own parameterisation. Two different types of report can be produced: one including the charts as displayed in the *Model Outputs* panel and one containing the selected map. The values of the parameters that are defined by the user also appear in the generated documents.

## Discussion

We introduce a user-friendly online tool capable of quantifying the proportions of MDR/RR-TB at re-treatment attributed to different causal pathways. This interface will help policy-makers to better identify the pathways to drug resistance and to design tailored programs to fight drug-resistant TB. While the higher rates of MDR/RR-TB at re-treatment by comparison to new cases have long been recognised and reported by WHO, the pathways leading to such gaps and their implications for effective local control strategies have remained unexplored for too long. Our findings suggest that primary transmission of drug-resistant strains is the predominant pathway in most settings. This challenges the old dogma that drug resistance amplification as a result of poor quality or adherence to treatment was the main explanation for the high rates of MDR/RR-TB at re-treatment. It highlights the need to refocus TB control interventions towards solutions that are specific to the drivers of MDR/RR-TB in a particular location.

The modelling interface presented in this report will enhance the applicability of this finding and we believe that it provides an important “proof of principle” of how modelling approaches can be adapted to provide a user-friendly interface for assisting local decision making. In this instance it could assist countries to estimate the contribution of different pathways to the generation of drug resistant TB cases, allowing for better targeted and better contextualized interventions to be considered. For example, finding high proportion of MDR/RR-TB at re-treatment arising from pre-existing MDR/RR-TB primary transmission may lead to a response to universally test all new cases of TB for resistance, whereas a finding of acquired amplification of MDR/RR-TB being the dominant source may lead to examination of the failure/loss to follow-up rates of those with DS-TB. As a “proof of principle” this tool demonstrates how user-friendly interfaces can be developed to allow public health decision makers, at different levels of care, access to modelling data that is relevant to their local setting.

The platform allows our existing model to be used in combination with flexible parameterisation entered by the user. In addition to offering the possibility for alternate parameter sets from those employed in our previous study, this interface also allows estimates to be obtained for a broader range of settings, and in particular at local levels. Most importantly, this tool will allow users with no specific technical knowledge to use a mathematical model and to compute personalised estimates.

We chose not to include uncertainty around parameter values when building this online interface in order to maximise the tool’s simplicity and to make it accessible to the broadest possible audience. Nevertheless, the users will be able to assess the sensitivity of the model estimates to the different parameters by using the different input sliders and produce outputs related to alternate parameter sets. In contrast, our previous study incorporated uncertainty and sensitivity analyses that highlighted the influence of some parameters on the contributions of the respective pathways [5]. In particular, we demonstrated that the contribution of inappropriate diagnosis to the burden of MDR/RR-TB at re-treatment was significantly affected when varying the rate of drug susceptibility testing or the parameters that relate to treatment outcomes (success and death rates). Further, we noted that the contribution of the pathway involving drug resistance amplification increased when reducing the treatment success rate for DS-TB and the individual-level risk of drug resistance amplification in unsuccessfully treated patients. Other conceptual assumptions than those considered in the baseline analysis may also be explored using the sliders. For example, patients who are lost to follow-up are assumed to experience unsuccessful treatment under the default behaviour of our model but alternate scenarios may be considered by increasing the parameter values that define the treatment success rates.

The web-based tool addresses some of the limitations of the original model, especially regarding the level of resolution of the analysis and the uncertainty around parameter values and underlying assumptions. However, some limitations that are linked to the intrinsic structure of the model could not be addressed here. For example, only two phenotypes of TB are considered in the model – DS-TB and MDR/RR-TB – although we know that other resistance profiles exist. This simplification was made in order to make the model broadly applicable and to match with the approach used by WHO to classify drug-resistant TB. Another limitation is that some more specific causal pathways such as nosocomial transmission of MDR/RR-TB are not considered in the model although there is evidence of their importance in some settings [11, 12]. More specific model structures may be designed to investigate the causes of MDR/RR-TB among re-treatment cases in such contexts. It is also important to note that the estimates provided by our interface are relative contributions of the different pathways expressed as percentage of the total burden of MDR/RR-TB at re-treatment. Therefore, the overall absolute burden of MDR/RR-TB at re-treatment should also be taken into account by users if they wished to estimate the absolute number of cases that are attributed to each of the four causal pathways.

User-friendly tools such as ours can contribute to building confidence in the use of modelling, which is essential to facilitate the bidirectional exchange between modellers and TB control program developers and improve the understanding of the local epidemic. We believe that this user-friendly adaptation presents an important exemplar of how modelling approaches could become more useful to guide everyday decision making processes using local data inputs for optimal contextualisation.

## Abbreviations

DS-TB: Drug-susceptible tuberculosis
MDR/RR-TB: Multidrug-resistant or rifampicin-resistant tuberculosis
TB: Tuberculosis
WHO: World Health Organization
